# Report of multiple high-grade gliomas in two patients with shared retained ATRX, wild-type IDH, losses of CDKN2A genes and alterations in the PTEN–PI3K axis

**DOI:** 10.1093/jscr/rjad139

**Published:** 2023-03-30

**Authors:** Christopher Khatchadourian, Jin Guo, Chaya J Prasad, Robert A Orlando, Cyrus Parsa

**Affiliations:** College of Osteopathic Medicine of the Pacific, Western University of Health Sciences, Pomona, CA 91766, USA; College of Osteopathic Medicine of the Pacific, Western University of Health Sciences, Pomona, CA 91766, USA; Department of Pathology, Beverly Hospital, Montebello, CA 90640, USA; College of Osteopathic Medicine of the Pacific, Western University of Health Sciences, Pomona, CA 91766, USA; Department of Pathology, Beverly Hospital, Montebello, CA 90640, USA; College of Osteopathic Medicine of the Pacific, Western University of Health Sciences, Pomona, CA 91766, USA; Department of Pathology, Beverly Hospital, Montebello, CA 90640, USA

**Keywords:** multiple gliomas, high-grade gliomas, glioma molecular tumorigenesis, glioma upgrades

## Abstract

Solitary gliomas have been well described in the literature. Multiple gliomas, however, have not received the same notoriety, and as such further studies may be helpful in elucidating their unique clinicopathologic features and molecular basis. We present two patients, each with multiple high-grade gliomas, and describe their clinicopathologic and molecular characteristics in comparison with those reported in the literature in an attempt to better understand their shared tumorigenic mechanisms. Extensive molecular, FISH and genomic profiling studies detected multiple unique abnormalities in our two cases with shared molecular features of retained ATRX, wild-type IDH, losses of CDKN2A genes and alterations in the PTEN–PI3K Axis.

## INTRODUCTION

High-grade gliomas (HGGs), such as glioblastoma (GBM), most commonly occur as a primary disease, typically in older individuals. Less frequently, however, these HGGs occur in younger patients as a secondary disease due to progression of a lower-grade astrocytoma. The latter tumors are often associated with driver mutations of mutant Isocitrate dehydrogenase 1 (IDH1), or its homologue IDH2. In contrast, most primary HGGs with or without histopathologic glioblastoma features are wild-type IDH, and harbor other genetic alterations, most commonly gains of chromosome 7, loss of chromosome 10, TERT-promoter mutations and EGFR gene amplification [1]. These and other genetic and genomic alterations, especially homozygous and hemizygous losses involving 9p21, have been observed at a high frequency in infiltrating gliomas. One of the consequences of 9p21 deletion is the loss of the cyclin-dependent kinase inhibitor CDKN2A gene, which results in cellular proliferation and dysregulation of pro-apoptotic pathways [2].

The PI3K–AKT–mTOR signal transduction pathway regulates a variety of biological processes including cell growth, cell cycle progression and proliferation, cellular metabolism and cytoskeleton reorganisation. Activating mutations of the p110α subunit of PI3K (PIK3CA) have been identified in a broad spectrum of tumors [3]. Inactivation of the tumor suppressor phosphatase and tensin homologue deleted on Chromosome 10 (PTEN) and/or activating mutations in the proto-typical lipid kinase PI3K have emerged as some of the most frequent events associated with human cancer. As a result, the PTEN–PI3K axis seems to play an essential role in controlling cellular behaviors by modulating the activation of key proto-oncogenic molecular nodes and functional targets [4].

A systematic review of multiple gliomas and meta-analysis of literature found the prevalence of multiple GBM among HGG patients to be 19%, of which the ages at presentation ranged between 28 and 86 [5], encompassing the two cases in this report. In a retrospective study of 7 patients with biopsy-proven multiple GBMs, the age of the patients upon presentation ranged from 17 to 69, with a mean age of 45 years [6]. All patients in the latter study were diagnosed with symptoms of elevated intracranial pressure (ICP), with variable impaired speech, impaired memory, headache and altered sensorium.

Immunohistochemical (IHC) staining performed on multicentric gliomas of 14 patients [7] revealed all to be negative for mutant IDH1. IHC staining further revealed that all but one of the patient’s tumors to be negative for nuclear phosphate and tensin homologue (PTEN). Eipdermal growth factor receptor (EGFR), p53 and alpha-thalassemia mental retardation X-linked (ATRX) showed positive IHC staining in 64.3, 35.7 and 28.6% of patients, respectively [3]. Fluorescence *in situ* hybridization, performed on nine of the specimens, showed no co-deletion for 1p/19q. Comparative genomic hybridization of left and right cerebral glioblastomas, reported as a case report in a 65-year-old man, revealed IDH-1 wild type with a p53 mutation at the same locus. Many of the same mutations were reported to be present in both tumors [8]. In a case-control study of 47 patients with multiple GBMs, 2-year survival was significantly decreased in the multiple GBM patients compared with those with solitary tumors [9].

In this study, we present two cases with multiple HGGs with extensive molecular, FISH and genomic profiling studies. The following pertinent findings were detected in our two cases: first case::wild-type IDH, MGMT Promoter Methylation, MET amplification, PDGFRA amplification, CDKN2A deletion and PTEN deletion; second case: wild-type IDH, TERT promoter alteration; activating PIK3CA alterations; copy number loss of 9p21.3 (CDKN2A, CDKN2B); and amplification of EGFR. The results in these two patients show shared retained ATRX, wild-type IDH, losses of CDKN2A genes and alterations in the PTEN–PI3K axis.

### Case 1

A 79-year-old male with a long history of type 2 diabetes mellitus, hypertension and cerebrovascular accident (stroke) was doing well, until 1 year prior to admission, when he started getting increasingly confused, and forgetful with slight slurred speech. The patient had been referred as an outpatient to the neurologist, but had not been able to get an appointment. He was brought into the emergency department (ED) by his son when he woke up one day with confusion and slurred speech. History was obtained from the patient’s son. The patient had complained of burning in the mouth and hands, and was unable to provide any further history. There were no witnessed seizures, falls, head injury, fever, chills or other complaints.

**Figure 1:**
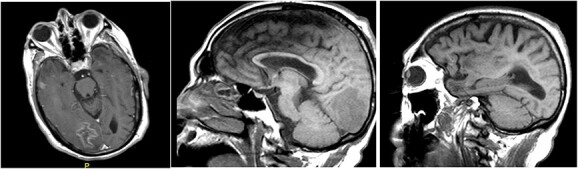
MRI of brain, with and without contrast, identified four enhancing lesions, concerning for intracranial metastatic disease, located in the right temporal (left), occipital (center) and frontal lobes (right).

Magnetic resonance imaging (MRI) of brain, with and without contrast, identified four enhancing lesions, concerning for intracranial metastatic disease, located in the right temporal, occipital and frontal lobes (Fig. 1), as well as one in the right cerebellum. The largest mass was in the right cerebellum and measured approximately 3 cm × 2.7 cm. There was associated edema throughout the right occipital and temporal lobes without significant mass-effect or midline shift. The patient’s chest, abdomen and pelvis CT were negative for any malignancy. Neurosurgery was consulted to perform a brain lesion biopsy.

**Figure 2:**
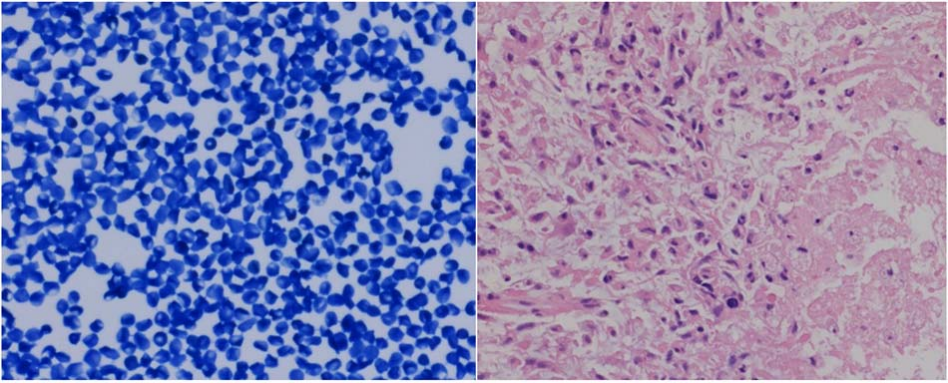
The tiny frozen section preparation confirmed the presence of malignancy, histologically reminiscent of lymphoma versus glioma. The permanent sections showed atypical glial proliferation with marked nuclear pleomorphism and necrosis (right corner of image).

Utilising intraoperative neuronavigation system, a tiny biopsy sample was obtained through a bur hole in the right occipital region, where the tumor appeared most superficial to the surface, and sent for frozen section/touch preparation and presumptive diagnosis. The touch preparation was morphologically reminiscent of lymphoma versus glioma (Fig. 2), requiring a review of the permanent sections and further studies for definitive diagnostic evaluation. Additional biopsies of the abnormal brain tissue were then submitted for permanent sectioning.

**Figure 3:**
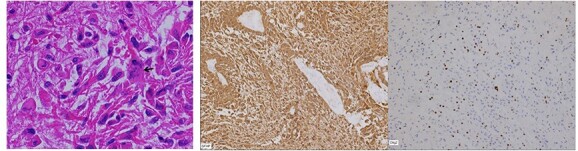
The permanent sections showed atypical glial proliferation with marked nuclear pleomorphism, increased vascularity and atypical mitoses (arrow in the left image). Immunostains were diffusely positive for GFAP (center image), and variably positive for Olig2.

The permanent sections of the abnormal brain tissue showed atypical glial proliferation with marked nuclear pleomorphism, increased vascularity, atypical mitoses and necrosis with diffuse GFAP IHC positivity, consistent with an HGG (Fig. 3). Additional IHC stains included positivity for IDH1, but negativity for Olig2 and p53.

Pertinent molecular, FISH and genomic profiling studies detected the following abnormalities: MGMT Promoter Methylation, MET amplification, PDGFRA amplification, PTEN deletion and CDKN2A (p16) homozygous deletion.

As pertinent negative findings, no abnormalities were detected in the following genes: 1p/19q Co-deletion FISH, ATRX, EGFRvIII, H3-3A, HIST1H3C, IDH1 and IDH2.

The presence of multifocal enhancing lesions on imaging, necrosis on histology and molecular studies showing wild-type IDHI as well as the loss of PTEN are consistent with the overall impression of a tumor behaving like a WHO Grade 4 glioblastoma.

### Case 2

A 57-year-old female with past medical history significant for depression, hypertension and type II diabetes mellitus was diagnosed with progressively worsening headaches and trouble finding words of one week duration. She complained of worsening progression of her depression and anxiety since 3 weeks. At the time of presentation, the patient had trouble speaking. She had no history of recent illness or sick contacts, and denied nausea, vomiting, changes in vision, abdominal pain, dysuria or diarrhea.

**Figure 4:**
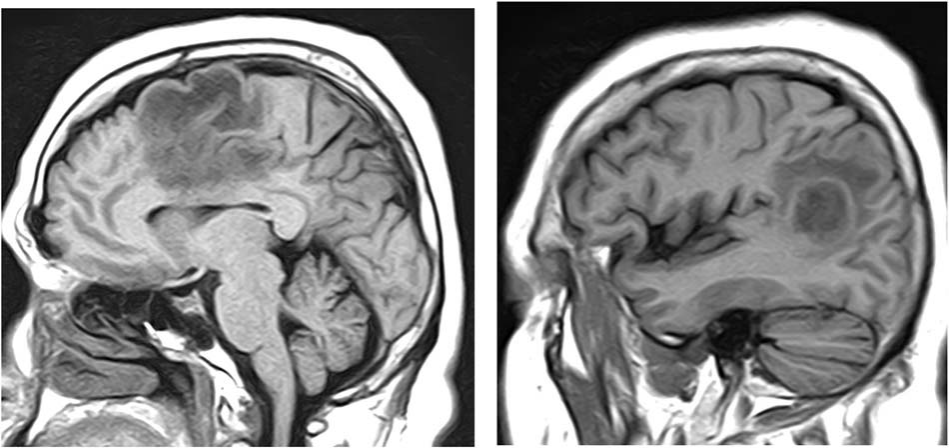
MRI of brain without contrast showed two enhancing masses in the left frontal and left parieto-occipital lobes, 2.1 cm × 2.7 cm × 1.9 cm and 1.9 cm × 2.0 cm × 2.5 cm, respectively.

The MRI of brain without contrast showed two enhancing masses in the left frontal and left parieto-occipital lobes (Fig. 4), 2.1 cm × 2.7 cm × 1.9 cm and 1.9 cm × 2.0 cm × 2.5 cm, respectively. These were interpreted as possibly representing the adjacent satellite lesions. There was extensive surrounding vasogenic edema, causing approximately 3 mm of the left to right midline shift. These enhancing left frontal and left parieto-occipital masses were interpreted as being compatible with brain metastases.

As the left frontal region was in the pre-central gyrus and near the falx, an open biopsy of the left parieto-occipital mass was considered the better neurosurgical approach. Several samples were sent from the border of the tumor for frozen section intraoperative presumptive diagnosis, as well as multiple samples from within the tumor for permanent sectioning and subsequent definitive histology diagnosis. Intraoperatively, there was no boundary between the brain and the tumor that would suggest a metastatic tumor.

**Figure 5:**
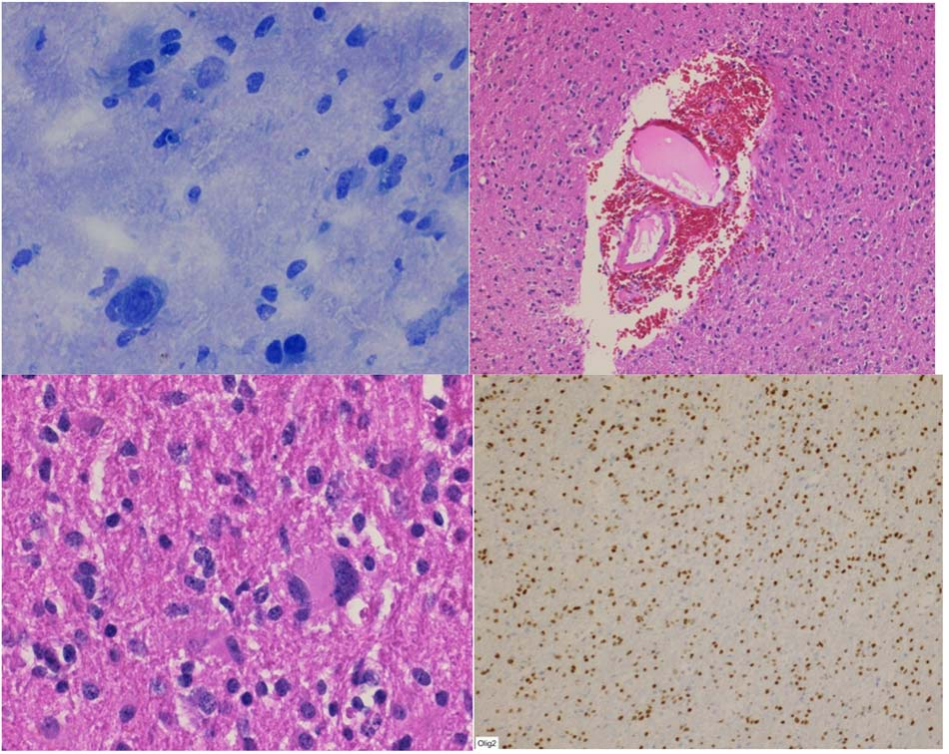
(**A**) Frozen section biopsy is consistent with a glial neoplasm. Permanent sections: (**B**) glial neoplasm with vascular endothelial proliferation; (**C**) nuclear pleomorphism; (**D**) Olig-2 immunostain positivity.

The frozen section specimens were consistent with a glial neoplasm (Fig. 5A). Permanent sections of the lesion showed glial proliferation consistent with diffuse astrocytoma (Fig. 5B and C). IHC stains were positive for GAFP, OLIG-2 (Fig. 5D), and retained ATRX. IDH1 R132H mutation and p53 were negative by IHC. Ki-67 was up to 10%.

Pertinent molecular and genomic profiling studies identified: TERT promoter alteration; activating PIK3CA alterations; copy number loss of 9p21.3 (CDKN2A, CDKN2B); amplification of EGFR; and negative MGMT promoter methylation.

According to the National Comprehensive Cancer Network (NCCN CNS cancers v2 2021) Guidelines, some diffusely infiltrating astrocytomas lack the histologic features of glioblastoma (necrosis, and/or microvascular proliferation), but have the molecular hallmarks of glioblastoma. Some features suggested as markers for subtyping grade II-III gliomas include the amplification of EGFR. In such cases, the tumor should be diagnosed as diffuse astrocytic glioma, IDH-WT, with molecular features of glioblastoma, WHO grade 4.

Gene variants of unknown significance identified in this case included the following: DSC3, INPP4B, NBN and POLE.

## DISCUSSION

The evolution of multiple gliomas, as described in the two patients presented in this paper, may implicate association of these rare tumors with some shared molecular and genomic features that warrant further studies. Amongst the four shared markers described in this paper, the Wild type IDH and PTEN-P13K alterations have been well studied in some prior publications (see below).

IDH1 mutations allow altered metabolite production that can be used as a prognostic marker to predict glioma response to radiation and alkylating chemotherapy [10]. IDH1 mutation in patients with GBM independently predicts greater survival compared with IDH1 wild-type GBM [11]. This may help explain the poorer prognosis in multiple gliomas discussed previously as they seem to have a propensity to express wild-type IDH1.

Multiple studies have shown that loss or modification of PTEN, a tumor suppressor gene whose deficiency causes homologous recombination deficits and upregulation of the PI3K/AKT/mTOR pathway in human tumors, can confer resistance against EGFR inhibitor therapy [12–14]. Expression of PTEN and EGFRvIII, a constitutively active mutant variant of EGFR, in malignant gliomas each confer greater sensitivity to EGFR inhibitor therapy [15]. On the other hand, the loss of homologous recombination consequent to PTEN expression loss in a tumor can render it more susceptible to poly-ADP-ribose polymerase (PARP) inhibitors [16]. Furthermore, PTEN-deficient tumors showed greater inhibition of growth when challenged with mTOR inhibitors [17], increased survival in response to Akt inhibitors [18] and decreased proliferation of tumors in response to combined therapy with PI3K and mTOR inhibitors [19]. Therefore, the loss of PTEN status may be helpful in constructing a treatment regimen for patients with multiple gliomas.

In summary, multiple gliomas are poorly understood tumors with an unfavorable prognosis compared with their unifocal counterparts. This poorer prognosis may be due to trends in tumor markers specific to multiple gliomas. Testing for retained ATRX, wild-type IDH, losses of CDKN2A genes and alterations in the PTEN–PI3K axis may provide further clinical aid in sub classification of lower-grade gliomas for their improved prognostic and therapeutic management. This, however, would require further elucidation with large scale studies to establish statistical significance.
